# Mining exceptional social behavior on attributed interaction networks

**DOI:** 10.1007/s10994-025-06831-z

**Published:** 2025-10-10

**Authors:** Martin Atzmueller, Carolina Centeio Jorge, Cláudio Rebelo de Sá, Behzad M. Heravi, Jenny L. Gibson, Rosaldo J. F. Rossetti

**Affiliations:** 1 Osnabrück University, Osnabrück, Germany; 2 German Research Center for Artificial Intelligence (DFKI), Osnabrück, Germany; 3 Tilburg University, Tilburg, The Netherlands; 4 University of Porto, Porto, Portugal; 5 University of Twente, Enschede, The Netherlands; 6 University College London, London, UK; 7 University of Cambridge, Cambridge, UK

**Keywords:** Subgroup discovery, Social interaction networks, Dyadic analysis

## Abstract

Social interactions are prevalent in our lives. These can be observed, e. g., online using social media, however, also offline specifically using sensors. In such contexts, typically time-stamped interactions are recorded, which can also be inferred from real-time location of humans. Such interaction data can then be modeled as so-called social interaction networks. For their analysis, a variety of different approaches can be applied. A prominent research direction is then the detection of patterns describing specific subgroups with exceptional behavioral characteristics, given some measure of interest. In the standard case of plain graphs modeling the interaction networks, methods for identifying such subgroups mainly focus on structural characteristics of the network and/or the induced subgraph. For attributed social networks, then additional attributive information can be exploited. This paper proposes to focus on the dyadic structure of the attributed social interaction networks, thus enabling a compositional perspective for identifying interesting subgroup patterns. Specifically, we can then analyze spatio-temporal data modeled as attributed social interaction networks for identifying exceptional social behavior. The presented approach adapts local pattern mining using subgroup discovery to the dyadic setting, exploiting attribute information of the spatio-temporal attributed interaction networks. With this, specific characteristics of social interactions are considered, i. e., duration and frequency, for identifying subgroups capturing social behavior that deviates from the norm. For subgroup discovery, we propose according interestingness measures in the form of seven novel quality functions and discuss their properties. In our experimentation, we perform an evaluation demonstrating the efficacy of the presented approach using four real-world datasets on face-to-face interactions in academic conferencing as well as school playground contexts. Our results indicate that the proposed method returns interesting, meaningful, and valid findings and results.

## Introduction

Identifying exceptional patterns that describe and capture interesting subgroups is a prominent research area in the context of social network analysis and mining e. g.,  (Burt, 1978; Wasserman and Faust, 1994; Frank, 1997; Atzmueller, 2014, 2015; Kong et al., 2019). Analyzing interactions in such networks allows us, e. g., to study human behavior in their role as social actors (Cabrera-Quiros et al., 2018). This concerns e. g., homophily (McPherson et al., 2001), i. e., a behavioral phenomenon of people to interact the more with people, the more those are similar to them.

In such contexts, automatic pattern mining (Atzmueller, 2018) for detecting unusual behavior can be valuable for enhancing the understanding of the according emerging set of interactions. For this, there are several application domains such as marketing (Delener, 1994), education (Altman, 1975), security (Ko, 2008), and health (Owen et al., 2010). Due to modern information technology, wearables and mobile computing, a large amount of behavioral data can be collected (Yu et al., 2019). In particular, sensors (e. g., proximity or geo-localization) enable the collection of rich behavioral sensor data of actors with limited or no interference (Heravi et al., 2018), such as movement data (Lauw et al., 2010), can be applied. Here, interaction information can then be modeled in the form of complex networks, i. e., *social interaction networks*, for capturing interactions between actors (Atzmueller, 2014). In addition, descriptive attributes can be added to nodes and/or edges, e. g., using socio-demographic or psycho-behavioral characteristics.

In this paper, an adapted and substantially extended revision of Atzmueller (2018) and Centeio Jorge et al. (2019), we propose a method for mining patterns of exceptional social behavior on attributed interaction networks. In such networks, nodes and/or edges are labeled with additional attributive information. This enables an extended analysis, by exploiting the further attribute dimensions for detecting patterns that describe specific subsets of nodes (subgraph) of the graph representation of a given social interaction network. While we build on the method proposed in Atzmueller (2018), we have, in particular, extended significantly on the set of quality measures, experiments and discussion on those, compared to Atzmueller (2018) and Centeio Jorge et al. (2019). In particular, we have considerably extended the experimentation. In our additional experiments, we also specifically discuss and demonstrate the applicability of specific families of quality functions, e. g., when assessing homophily, and demonstrate their specific impact on the analysis. This is also the major difference to our work presented in Centeio Jorge et al. (2021), where we focus on spatial outlier analysis. To summarize, we have specifically extended the paper in the following directions:

1. We present a new type of quality function regarding compositional subgroup discovery, specifically focusing on the phenomenon of homophily, which is instantiated in two novel quality functions. For these, we provide an integrated formalization of these quality functions and discuss their properties in detail.
2. We provide novel analysis results on the applied datasets and discuss them in detail, especially focusing on the new quality function that we propose.
3. Based on methodological considerations as well as our results, we discuss when to apply which kind of quality function for compositional subgroup discovery.
4. In addition, we provide an open-source implementation demonstrating the presented approach together with benchmark datasets.

*Problem* In this paper, we focus on the problem of discovering interesting compositional patterns describing/covering specific subsets of dyads in an attributed network. The patterns thus are formed using the attributive information, while they correspond to subgroups of edges given a graph representation of the attributed network. The presented methods are based on subgroup discovery (Atzmueller, 2015) which provides simple to interpret patterns to the user. Here, we specifically combine subgroup discovery, complex network analysis and network science methods for detecting patterns indicating unusual social interactions. For this, we build upon compositional subgroup discovery on such attributed social interaction networks (Atzmueller, 2018). For the extension towards social, spatial, and temporal characteristics, we present specific interestingness measures for estimating the quality of a subgroup, in order to find subgroups of people whose interactions deviate from the norm. For estimating the quality, we consider the structure of the respective subgraphs induced by a pattern. In our case, we consider attributed social interaction networks, where the edges (dyads) are labeled e. g., with the duration and frequency of the respective interactions. Here, the applied quality measure should consider those patterns as especially interesting which deviate from the expected “overall” behavior given by a null-model, i. e., one that models dyadic interactions due to pure chance.

*Objectives & Methods* Given an attributed social interaction network, our objective is to identify interesting patterns inducing a specific subgraph given by a subgroup of nodes; their interestingness is estimated according to their dyadic structure by a quality measure indicating interesting (social) behavior. Our presented novel approaches are based on core ideas of subgroup discovery and exceptional model mining (Atzmueller, 2015; Duivesteijn et al., 2016; Atzmueller et al., 2016) for which we demonstrate adaptations to network and graph representations. Further, we propose seven novel quality functions, that are statistically well-founded.

*Contributions* Our contribution is summarized as follows:

1. In a unified perspective, we formalize the problem of detecting exceptional social behavior via compositional subgroup discovery based on capturing interesting subgroups of dyads, in coherently contextualized discussion.
2. We accordingly discuss seven targeted quality measures for compositional subgroup discovery, of which three novel ones take social homophilic constraints and directed networks into account. The proposed quality functions are statistically well-founded, providing a statistical significance value directly.
3. In addition, we demonstrate the efficacy of our proposed approach and the presented quality measures using four real-world datasets capturing social face-to-face and socio-spatial interaction networks.
4. Finally, the release of our software package^1^ demonstrating the presented approach together with benchmark datasets is a major contribution of this paper.

*Structure* The rest of the paper is structured as follows: Section 2 discusses related work. Section 3 introduces and formalizes method and theoretical background. After that, Sect. 4 outlines the proposed approach. Next, Sect. 6 presents results of the proposed method analyzing four real-world datasets. Finally, Sect. 7 concludes with a summary and points out interesting directions for future work.

## Related work

Below, we summarize related work on complex (attributed) interaction networks, subgroup discovery, exceptional model mining, and developmental psychology.

### Modeling and mining complex interaction networks

A network is often modeled as a graph consisting of a set of *nodes* and a set of *edges* connecting the nodes. For example, social networks represent people or groups of people (nodes) and relationships between them (edges). Social interaction networks (Wasserman and Faust, 1994, p. 37 ff.) model *interaction* relations between actors represented by *people*, e. g., as to sensor-based face-to-face networks (Isella et al., 2011; Scholz et al., 2013). We can then empirically study such networks to gain an understanding of their underlying structure, e. g., regarding communities (Newman, 2006), or homophily (McPherson et al., 2001).

Attributed (or labeled) graphs enable approaches that specifically exploit the descriptive information of the labels assigned to nodes and/or edges of the graph (Interdonato et al., 2019). These edges may have properties, such as frequency of occurrence or duration. Furthermore, socio-spatial networks can also be derived using spatial properties providing information about objects’ location, e. g., exploiting information about *movement data* (Lauw et al., 2010).

For mining attributed interaction networks there exist a variety of techniques, including link prediction (Liben-Nowell and Kleinberg, 2007; Al Hasan and Zaki, 2011) and community detection (Newman, 2004; Bothorel et al., 2015). Link prediction can be defined as predicting edges to be added over time to a network. Regarding social interaction networks, there have been several approaches in the literature, e. g.,  (Liben-Nowell and Kleinberg, 2007; Taskar et al., 2003), also considering offline social interaction networks (Scholz et al., 2013, 2014). Extensions on attributed interaction networks specifically utilize the attribute information together with the network topology, e. g.,  (Al Hasan and Zaki, 2011; Gong et al., 2014; Guven et al., 2020). Community detection focuses on the detection of densely connected groups or clusters in a network, e. g.,  (Newman, 2004). Standard algorithms for community detection (Newman, 2004; Blondel et al., 2008; Rosvall and Bergstrom, 2008; Abbe, 2018) start on simple interaction networks, and do not make use of the additional (descriptive) information. Extensions (Bothorel et al., 2015; Combe et al., 2015; Falih et al., 2018; Atzmueller et al., 2021) on feature-rich and in particular attributed networks (Bothorel et al., 2015; Interdonato et al., 2019) make use of the rich attribute information of nodes and/or edges. In our context, specifically descriptive community detection algorithms are relevant – see (Atzmueller et al., 2021) for a survey. Here, the basic idea is that attribute information of the attributed networks is applied such that each community is given as a set of nodes together with a *description* in terms of the attributive information - in order to serve as an interpretable representation.

In contrast to those approaches, we do not focus on detecting connected groups or clusters, optimizing the respective quality functions there, but take a dyadic perspective on the data, in order to identify subgroups of dyads with a specific description. In addition, we consider specific quality functions beyond mere (weighted) connectedness, density, etc. as done in classical community detection.

### Subgroup discovery and exceptional model mining

Subgroup discovery (Atzmueller, 2015) focuses on detecting interesting subgroups induced by descriptive relations between a concept of interest and a set of explaining features. The applied interestingness criteria are formalized by a quality function (also called quality measure) ranking subgroups, e. g.,  (Herrera et al., 2011; Atzmueller, 2015) via target properties. Those target properties can be, e. g., binary (Wrobel, 1997), nominal (Berlanga et al., 2006), numeric (Grosskreutz and Rüping, 2009), ranked (de Sá et al., 2016), distributional (Jorge et al., 2006), sequential (Ribeiro et al., 2024) or they can consider complex graph structures (Atzmueller et al., 2016). For the latter, a quality measure can, for example, consider the density of a subgroup induced by a pattern, in relation to a null-model (Atzmueller et al., 2016). Exceptional model mining (Duivesteijn et al., 2016) focuses on such complex target properties, e. g., for outlier detection (Riahi and Schulte, 2020), or (spatio-)temporal analysis (Bueno et al., 2020; Du et al., 2020; Mollenhauer and Atzmueller, 2020; Schouten et al., 2022). In this work, we adopt the definition in Morik (2002); Atzmueller (2015), such that subgroup discovery can also be considered as having exceptional model mining as a special case, thus bridging both approaches.

In contrast to the approaches discussed above, our presented method adapts subgroup discovery for dyadic analysis: We focus on parameters such as the respective interaction *frequency* and *duration*. Based on previous work (Atzmueller, 2018; Centeio Jorge et al., 2019, 2021), we extend this towards socio-spatial and temporal characteristics in a unified approach. To the best of the authors’ knowledge, no other subgroup discovery approach tackling this problem has been proposed so far. We further present according novel quality functions in the socio-spatial/dyadic setting, also taking into account homophily which is particularly important in the context of social interaction networks. The proposed quality functions also ease statistical interpretation by providing a significance value directly.

### The potential application to developmental psychology

Although network-based approaches to understanding children’s social relationships have a long history in the literature, e. g., Rubin et al. (2011), it is only recently that researchers have turned to sensor-based methods (Messinger et al., 2019; Gibson et al., 2018; Veiga et al., 2017). Further, to the best of the authors’ knowledge, subgroup discovery methods such as those proposed in the current paper have not previously been used in those contexts.

The proposed subgroup discovery methods have the potential to greatly enhance current understanding of the formation and composition of friendships and relationships as they are enacted in children’s everyday interactions. For example, it is reasonably well established that children who have fewer friendships, and who are less well-liked by peers tend to be at risk of poor outcomes in domains as diverse as health, academic achievement and subjective well-being  (Rubin et al., 2011). However, using traditional observational methods it is difficult to gain insight into how social relationships unfold during everyday interactions and to understand how individual attributes of each child contribute to the construction of friendship groups. Using subgroup discovery methods, we aim to gain insight into significant patterns of affiliative behaviours.

## Method

Below, we briefly review relevant background on subgroup discovery, before we introduce according basic notions of our compositional network analysis approach.

### Background on subgroup discovery

Below, we partially follow the notation and formalization presented by Atzmueller (2018): For a set of attributes *A*, for each attribute $a \in A$ (e. g., *age, conference track* etc.), a range ${dom}(a)$ of attribute values is defined. We call an attribute/value assignment $a = v$, where $a \in A, v \in \textit{dom}(a)$, an *atomic selector*, e. g., *conference track* $=$ *1*. $V$ denotes the (universal) set of all atomic selectors. Generalizing to sets of atomic selectors, we call a selector $s \subseteq \textit{dom}(a)$ an extended selector, or a selection expression. *S* denotes the set of all selection expressions.

An *edge–attributed database* $\textit{DB}=(E, A, F)$ is given by a set of edges *E* and a set of attributes *A*. The edge–attributed database can be extracted, for example, from an edge-attributed graph/interaction network. For each edge $e \in E$ there is a mapping $F: E \rightarrow 2^{V}$ describing the set of selectors that are assigned to an edge.

A *pattern* describes a *subgroup*, i. e., the subgroup consists of the edges (and the respective nodes) that are covered by the respective pattern, i. e., those having the respective set of selectors. It is easy to see, that a pattern describes a fixed set of edges (inducing a subgroup of nodes), while a subgroup can also be described by different patterns, if there are different options for covering the subgroup’ edges. A (subgroup) *pattern* $\textit{P}$ is defined as a conjunction $\textit{P}= s_1 \wedge s_2 \wedge \dots \wedge s_n\,,$ of (extended) selectors $s_i \in S$. In general, a selection expression *s* is a Boolean function $E \rightarrow \{0,1\}$ that is true if the value of the corresponding attribute is contained in the respective subset of $V$ (and *S*, respectively) for the respective edge $e \in E$. Typically, atomic selectors $s \in V$ are used, while selectors $s \in S$ in general allow internal disjunctions via selecting subsets of values of an attribute.

A *subgroup* – in our case a set of edges $\textit{E} _{\textit{P}} \mathrel {\mathop {:}}=\textit{ext}(\textit{P}) \mathrel {\mathop {:}}=\{e \in E | \textit{P}(e) = \textit{true}\}$ is the set of all edges which are covered by the pattern $\textit{P}$. Using that, the induced subset of covered nodes can also be extracted.

A subgroup quality function $q :2^S \rightarrow \mathbb {R}\,.$ determines the interestingness of a subgroup. The quality function maps a pattern (equivalently its extension, i. e., the induced edge-set) to a real number based on specific properties of the pattern and/or its extension, respectively. For top-*k* subgroup discovery, the best *k* patterns as ranked by the applied quality function are given as a result. Alternatively, also a minimal quality threshold can be applied. In the simplest cases, i. e., for binary or numerical targets, typical parameters include the size $n = |ext(\textit{P})|$ of a subgroup and its deviation $t_{\textit{P}} - t_0$, where $t_{\textit{P}}$ is the average value of a given target variable in the subgroup identified by the pattern $\textit{P}$ and $t_0$ the average value of the target variable in the whole dataset. According standard quality functions are of the form, trading-off size and deviation $q_a (\textit{P})= n^a \cdot (t_{\textit{P}} - t_0),\, a \in [0;1]\,.$ Examples include so-called *simplified binomial* function $q_{a}^{0.5}$ for $a=0.5$, or the *gain* quality function $q_a^0$ with $a=0$. While such formalizations are utilized by standard subgroup discovery approaches, these do not cover the specific properties which are necessary for dyadic network analysis. Therefore, we provide specific adaptations for this case below. In addition, while a quality function provides a *ranking* of the discovered subgroup patterns, often also a statistical assessment of the patterns is useful in data exploration. Quality functions that directly apply a statistical test, e. g., the Chi-square quality function (Atzmueller, 2015) provide a *p*-value for simple interpretation. For network data, often randomization approaches can be used for estimating statistical significance, e. g., by comparing a network structure to a null-model. Approaches for community detection can also be adapted as quality measures in subgroup discovery, cf.  (Atzmueller et al., 2016). Another standard metric used in network analysis is the quadratic assignment procedure (Krackhardt, 1987) (QAP), as a graph correlation measure: For comparing two graphs $G_1$ and $G_2$, it estimates the correlation of the respective adjacency matrices $M_1$ and $M_2$ and tests that graph level statistic against a QAP null hypothesis (Krackhardt, 1987). The according null model (and its respective distribution) is given by repeated random row and column permutations of the adjacency matrix of $G_2$. However, it is important to mention, that this relates to the correlation of the whole graph and not to specific subgroups of dyads/edges.

As presented in Atzmueller (2018), we can devise quality functions based on similar considerations. Essentially, we need to compare a sub-network induced by a given subgroup pattern with a set of randomized sub-networks given the same distributional characteristics with respect to the total set of edges, taking into account specific characteristics of the expected number of edges given a null-model.

### Compositional network analysis using subgroup discovery

In this paper, we summarize and present approaches for analyzing dyadic interactions, i. e., those occurring between two “actor” nodes represented by edges in an according graph, cf.  Atzmueller (2018). We also investigate interaction networks constructed from spatial data, resulting in socio-spatial interaction networks, as a special case. Given *attributed* social interaction networks, we furthermore consider properties of nodes/and or edges, e. g., given by socio-demographic or psycho-behavioral characteristics of the involved actors, which can be further enriched using network science methods for inferring further labels. These are then used to characterize subgroups for *explaining* a certain (observed) behavior, e. g., Wasserman and Faust (1994); Frank (1997); Lau and Murnighan (1998), where subgroups are induced by (a set of) describing attributes.

As we have presented in Atzmueller (2018), we distinguish between the following properties for defining according quality functions focusing on numeric target features $t_P$. In the simplest case (unweighted), this corresponds to the observed number of edges normalized by the expectation, for pattern *P*. For ranking subgroups, we utilize the (normalized) mean of that target feature $t_P$.

1. Interaction duration: In social interaction networks, the duration of an interaction can be captured by a weight assigned to a specific link connecting the interacting actors. Then, simple networks that just capture those interactions can be represented by weighted graphs. In the unweighted case, we can just assign a default weight *w* for an edge *e*, e. g., $w(e) = 1.0$.
2. Interaction frequency: The frequency of interactions is typically indicated by multiple links between the two interacting actors, represented by a set of edges connecting the respective nodes in a multigraph. In addition, the duration of the interaction can also be captured as described above.

### Quality measures

Below, we put our analysis of social interactions into the context of according quality measures. With these, we provide important background relating to the applied quality measures for compositional subgroup discovery. Below, we summarize key results from Atzmueller (2018); Centeio Jorge et al. (2019) providing an extended contextualization of the proposed approach. We discuss four quality functions for the analysis of the social interaction networks, following our presentation in Atzmueller (2018); Centeio Jorge et al. (2019) for estimating *dyadic means* of a pattern *P*, corresponding to a numeric target feature $t_P$ with respect to a graph structure. We start with simple attributed non-directed graphs and multigraphs, which can also be weighted. Then, we present formalizations for digraphs and multidigraphs. Finally, as described in Atzmueller (2018), for all quality functions, we present randomization approaches to estimate statistical significance.

#### Attributed simple graph

In the case of a simple undirected network we can simply add up the number of (weighted) edges $E_P$ captured by a pattern *P*, and normalize by the number of all possible edges $n_E$ in the node subset induced by *P*. Thus, for a pattern *P*, we estimate its quality $q_{S}$(P) as $q_{S}(P) = Z(\frac{1}{n_E} \cdot \sum \limits _{e \in E_P} w(e)),$ with $n_E = \frac{n_{E_P}(n_{E_P}-1)}{2}$, where $n_{E_P}$ is the number of nodes covered by a pattern *P*. *Z* is a function that estimates the statistical significance of the obtained value (i. e., $t_P$) given a randomized model, which we discuss below in more detail.

#### Attributed multigraph

Attributed multigraphs allow the modeling of multi-links between actors. In particular, for normalizing the mean of target $t_P$, we also need to take into account the multiplicity of edges between the individual nodes.

With $n_E = \frac{n_{E_P}(n_{E_P}-1)}{2}$ indicating the total number of (single) edges between the individual nodes captured by pattern *P*, $m_i, i = 1 \ldots n_E$ models the number of multi-edges for an individual edge *i* connecting two nodes. Extending the simple graph case above for a pattern *P* to the multigraph case, we estimate its quality $q_{M}(P)$ as follows: $q_{M}(P) = Z(\frac{1}{n_E + m_E} \cdot \sum \limits _{e \in E_P} w(e))\,$ with $m_E = \sum \limits _{i=1}^{n_E}(m_i - 1)$.

#### Attributed simple digraph

The quality measures $q_{S}$ and $q_{M}$ are designed for non-directed networks. Extending undirected graphs to directed ones regarding interactions allows us to use the directionality information for characterizing the respective social behavior and according interactions. In particular, this means, for example, that we can distinguish between an interaction from *A* to *B* in contrast to one from *B* to *A*, where *A* and *B* correspond to specific actors which are represented by nodes in a graph.

For directed simple graph structures, i. e., digraphs, the quality measure $q_{\textit{DS}}$ is defined as: $q_{\textit{DS}}(P) = Z(\frac{1}{n_E} \cdot \sum _{e \in E_P} w\left( e\right) ) ,$ where $n_E = n_{Ep}(n_{Ep} - 1)$. Thus, in the directed case, the number of possible edges is twice as big as for $q_{S}$.

#### Attributed multidigraph

As for the undirected case, attributed directed multigraphs (multidigraphs) allow us to model multiple interactions between the same set of actors (nodes) in the directed case. Extending the measure $q_{M}$ above for multidigraphs yields $q_{\textit{DM}}(P) = Z(\frac{1}{n_e + m_E} \cdot \sum _{e \in E_P} w\left( e\right) ) ,$ where $n_E = n_{Ep}(n_{Ep} - 1)$ and $m_E = \sum _{i=1}^{n_E}(m_i - 1)$ and $m_i$ is the observed multiplicity of an edge. In this case, for modeling social interactions one directed edge is created every time an interaction is observed.

#### Randomization-based significance estimation

As outlined above, standard quality functions for subgroup discovery typically compare the mean of a given target concept with the mean estimated in the whole dataset. However, in the dyadic analysis that we tackle in this paper – referring to our presentation in Atzmueller (2018); Centeio Jorge et al. (2019) in the following – we also need to take edge formation of dyadic structures into account, such that, e. g., simply calculating the mean of the observed edges normalized by all edges for the whole dataset is not sufficient. In addition, since we use subgroup discovery for identifying a dyadic subgraph (i. e., a set of edges) induced by a pattern, we also aim to confirm the *impact* by checking the statistical significance compared to a null-model. For that, we propose a sampling based procedure: We draw *r* samples without replacement with the same size of the respective subgroup in terms of the number of edges, i. e., we randomly select *r* subsets of edges of the whole graph. According to the proposed quality functions, we distinguish four different cases:

1. $q_{S}$: In the simple graph representation, we just take into account the $N = \frac{n(n-1)}{2}$ possible edges between all nodes of the simple graph. Thus, in a sampling vector $R = (r_1, r_2, \ldots , r_N)$, we fill the $r_i, i = 1 \ldots N$ positions with the weights of the corresponding edges of the graph, for which that a non-existing edge in the given graph is assigned a weight of zero.
2. $q_{M}$: In the multigraph case we also consider the number of all possible edges between all the nodes, however, we also need to take the multi-edges into account, as $N = \frac{n(n-1)}{2} + \sum \limits _{i=1}^{n}(m_i - 1)\,,$ where $m_i, i = 1, \ldots , n,$ are the respective multi-edge counts for an individual edge *i*. As above, we assign the sampling vector *R* accordingly, and set the weight entries of non-existing edges to zero.
3. $q_{\textit{DS}}$: In the simple (observed) digraph case, we just take into account the $N = n(n-1)$ possible edges between all nodes of the simple digraph.
4. $q_{\textit{DM}}$ (observed multidigraph representation): $N = n(n-1) + \sum \limits _{i=1}^{n}(m_i - 1)\,.$

For selecting the random subsets, we apply sampling without replacement. This is essentially similar to a shuffling based procedure, e. g.,  (Duivesteijn and Knobbe, 2011; Gionis et al., 2007)). Then, we determine the mean of the target feature $t_R$ (e. g., mean duration) in those induced *r* subsets of edges, i. e., the *r* samples. Using the mean $t_P$ in the original subgroup and the set of *r* sample means, we can construct a z-score which directly leads to statistical assessment for computing a p-Value. This is modeled using the function $Z(t_P), Z: \mathbb {R}\rightarrow \mathbb {R}$ which is then used for estimating the statistical significance of the target $t_P$ of pattern *P*. In order to ensure that the *r* samples are approximately normally distributed, we can apply a normality test, for example, the Shapiro-Wilk-test (Shapiro and Wilk, 1965). If normality is rejected, a possible alternative is to compute the empirical p-value of a subgroup (Gionis et al., 2007). However, in practice often the distribution of the sampled means is approximately normally distributed, so that a p-value can be directly computed from the obtained z-score.

### Spatio-temporal behavior analysis using subgroup discovery

As presented in Atzmueller (2018); Centeio Jorge et al. (2019) spatio-temporal behavior analysis using subgroup discovery can be implemented building on compositional subgroup discovery, in order to discover exceptional behaviour, i. e., social behaviour which deviates from the norm. For that, we transform spatio-temporal data into socio-spatial interaction networks, and enrich those with network science metrics and structural concepts (see below). We can further distinguish between undirected and directed graphs using spatio-temporal behavior analysis, in particular when we can utilize movement data for inferring directionality information.

#### Integrating movement data with social data

Regarding the spatial data, the proposed approach essentially integrates *movement data* with *social data* of the actors. The respective *movement data* consists of timestamped data, i. e., *ID* and position (*x* and *y*) for each actor. Then, we can compute speed, *velX* and *velY*, relative to *x* and *y*. Regarding the according social properties, this includes socio-demographic data of the respective actors, where any numeric attributes are discretized in equal frequency bins. From movement data we can extract the directions of the interaction, meaning that we are able to know if one actor reached another one. This leads us to two possible interaction networks’ structures: directed or non-directed, either as a simple digraph or as a multi-digraph, with multiple edges between nodes.

#### Creating digraphs using movement data

For creating the respective digraphs or multidigraphs, we consider an interaction between two actors depending on their relative distance: if it is within a certain proximity – according to a maximum distance threshold *maxdist* – and one of the subjects approaches the other, then we define this as an interaction. We start with an empty digraph *G*. Then, we proceed as follows:

1. *Distance matrix*: For each time step *t*, we calculate a distance matrix *D* for all pairs of subjects (*i*, *j*).
2. *Distance vector*: For each respective distance $d_{i,j} \in D: d_{i,j} \le maxdist$ we compute a vector from *i* to *j* as $\vec {r}_{i,j} = \left( x_j-x_i,y_j-y_i\right)$.
3. *Directionality*: We then verify the speed vector of *i*, $\vec {vel}_i = (velX_i,velY_i)$, and calculate the cosine between the vectors $\vec {r}_{i,j}$ and $\vec {vel}_i$. Should the cosine be positive, we consider that the subject *i* *approached* (or *reached*) subject *j*. In this case, we create an edge as follows:
  Simple digraph case: a directed edge from node *i* to node *j* is added to *G*; $w_{i,j} \in W$ is incremented by one unit of time, where *W* is the matrix of weights and $w_{i,j}$ is the number of times subject *i* approaches subject *j*.
  Multidigraph case: a directed edge is added to *G* at moment *t* if subject *i* approached subject *j*, given that it was not interacting in $t - 1$, with $w_{i,j} \in W$. where $w_{i,j}$ is the total time that the subject *i* approaches the subject *j* without interruption.

### Generating edge attributes

For compositional subgroup discovery work, we can also generate edge attributes, cf.  (Atzmueller, 2018; Centeio Jorge et al., 2019), for which we summarize the relevant background in the following. As outlined in Atzmueller (2018), we consider edges connecting two nodes – corresponding to the respective actors that are involved in the interaction. For describing those interaction edges, we consider the assigned properties of the nodes. Consequently, the attributes of the edges are based on the comparison of the values of the respective nodes’ attributes. Accordingly, we create features on the edges of the attributed (multi-)graphs in such a way, so that an edge is labeled with and *feature-equal condition*, i. e., “<feature>=EQ”, if the respective nodes share the same value of a feature, e. g., *gender=female* for both nodes. Otherwise, the edge is labeled with a *feature-not-equal condition*, i. e., “<feature>=NEQ”.

*Example:* With the procedure described above, for example, the subgroup described by the pattern *gender=EQ* contains the nodes, for which the dyadic actors always agree on their attribute *gender*. Equivalently, for edge (*a*,*b*), and attributes *Gender* and *Age*: if *a*’s attributes are “*Gender*=F, *Age*=0"and *b*’s attributes are “*Gender*=M, *Age*=1", then we consider the edge (*a*,*b*) having the attributes “*Gender*=NEQ, *Age*=NEQ", because the nodes do not share either of the attributes’ values.

*Extension for directed graphs:* For directed (multi-)graphs, we propose three further options to assign attributes to edges, cf.  (Centeio Jorge et al., 2019):

- **Comparison:** Here, the attributes are compared in the direction of the edge. If the attributes are nominal, we create a tuple containing both values; if they are numerical, we compare them resulting in either “same", “higher"or “lower". Following the second example above, we obtain: “*Gender*=(F,M), *Age*=higher"
- **To-Node:** For that, the edge’s attributes are the same as the starting (or *head*) node. Following the example given above, we obtain: “*Gender*=M, *Age*=1"
- **From-Node:** Here, the edge’s attributes are the same as the ending (or *tail*) node. Following the example given above, we obtain: “*Gender*=F, *Age*=0"

With these variants we can find valuable information about the attributes of the subjects that look for interactions (*From-node*) and the subjects that are reached the most (*To-node*).

*Inclusion of network science metrics:* Since some datasets originally do no contain many attributes for description, we also propose to generate attributes using complex networks’ metrics, i. e., (1) (in-/out-)degree, (2) centrality measures (i. e., betweenness, closeness, eigenvector centrality), (3) as well as authority and hub values. For a formal discussion and introduction of those metrics we refer to standard literature, e. g.,  (Wasserman and Faust, 1994; Newman, 2010). These can then increase the range of possible patterns and may lead to conclusions regarding the structure of the network or characteristics of the node (e. g., a high betweenness value may suggest a high popularity).

## Balanced quality measures for social interaction analysis

**Fig. 1:**
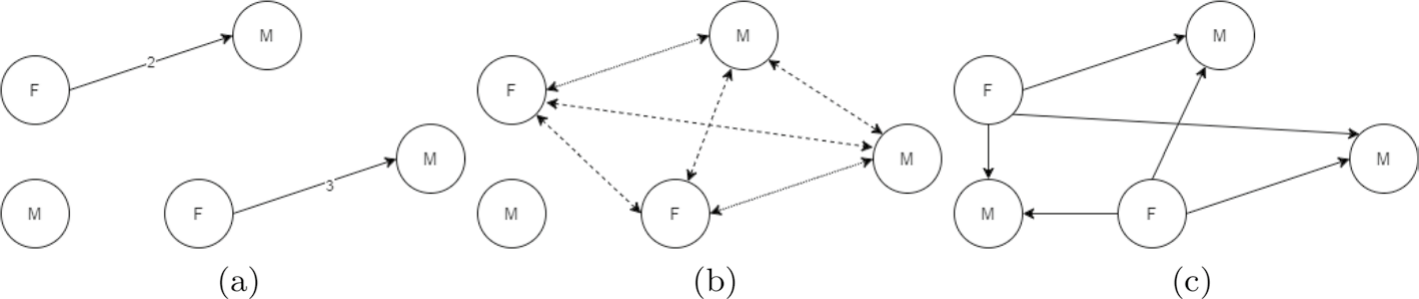
Homophily-aware pattern analysis: **a** interactions between 4 nodes, **b** edges in computation of $q_{\textit{DS}}$ (all possible edges that involve the nodes taking part in the interaction), and **c** edges in computation of $q_{\textit{BD}}$ (all possible edges in the complete graph, with a label that is compatible with the pattern/direction)

When analyzing dyadic interactions, we focus on interactions occurring between two “actor” nodes represented by edges in an according graph, cf.  Atzmueller (2018), in relation to a given null model. So far, it is important to note, that we use the number of all possible contacts (edges) for computing the mean of $t_P$, i. e., including edges with a zero weight. However, there is a special case to be potentially considered when computing the set of the possible edges. We can only consider those possible edges as the edges that can be *covered by a specific pattern* – in contrast to *all possible* edges. This is especially important when analyzing social interaction networks. In more detail, we distinguish whether we normalize the sum of the dyadic target values relative to the *complete subgroup* of nodes induced by a pattern, and all their possible interactions, or to all the *possible dyads* between nodes of the subgroup induced by a pattern, i. e., all dyads that are possible according to the pattern description (and its semantics). That is, we can either count all possible edges in the interaction subgraph, potentially counting edges twice, which could bias homophily. Alternatively, we can correct for the directionality and the edges which can be covered by a pattern. In the latter case, e. g., considering the attribute *gender*, with the set of values *(female, male)* and the pattern *gender=NEQ*, we would only consider interactions between *female–male*. An example is shown in Fig. 1 for what the analysis would be when considering edges that go from a *female* node to a *male* node.

This approach enables two complementary perspectives: First, we can consider the complete subgroup of nodes that is induced by a specific pattern, i. e., the set of nodes linked via dyads covered by the pattern. Then, we can compare the target feature $t_P$ in the pattern relative to all possible (unrestricted) interactions between those nodes. This also enables to compare the mean and variance across subgroups with their respective sizes more easily. Second, we can compare the target feature $t_P$ in the pattern relative to all possible interactions restricted to all the possible dyads according to the pattern, also for investigating specific hypotheses, e. g., homophily, in more detail. In the following, we call the latter quality measures *balanced quality measures*, in contrast to *simple* ones. Finally, we can also consider variants in signed graphs.

### Attributed balanced (multi-)graph

In contrast to the quality measure presented above, we can also focus only on the possible edges as the edges that can be covered by the pattern *P*. That is, for the normalization, we do not consider all possible interactions according to the vertices of the subgraph induced by the pattern, but we focus only on those interactions, that are possible to be covered by the pattern *P*. For example, in the case of the attribute *gender*, with value range *(female, male)* and the pattern *gender=EQ*, we would only consider interactions between *female–female* and *male–male*. As such, we consider the graph as *completed* (when an interaction between two nodes is not observed, the edge exists and has $weight=0$). Thus, for this quality measure, $E_{CP}$ is the set of edges that are covered by the pattern *P*, including observed ($weight>0$) and not observed ($weight=0$) interactions. Then, we normalize by the size of $E_{CP}$. Accordingly, the quality function, $q_{B}$, is formalized as follows:

1 $$\begin{aligned} q_{B}(P) = Z\left(\frac{1}{|E_{CP}|} \cdot \sum _{e \in E_{CP}} w\left( e\right) \right) \end{aligned}$$

It is important to note, that with this quality function the normalization of the mean values as the resulting quality value is always relative to the pattern semantics, not to the pattern size. Therefore, the quality values between patterns are not comparable according to the sizes anymore, but can yield interesting semantic interpretation, as we will also outline below.

### Attributed balanced digraph

As for the digraph measures described above, this quality measure also takes into account the duration of the interactions between two subjects in a digraph. However, in this version, we only consider the possible edges as the edges that can be covered by the pattern *P*, similar to measure $q_{B}$ discussed above. As such, we consider the graph as *completed* (when an interaction between two nodes is not observed the edge exists and has $weight=0$). Then, we normalize by the size of the possible edges (dyads) according to pattern *P*, as discussed above.

Quality function, $q_{\textit{BD}}$ is formalized as follows:

2 $$\begin{aligned} q_{\textit{BD}}(P) = Z\left(\frac{1}{|E_{Sp}|} \cdot \sum _{e \in E_{Sp}} w\left( e\right) \right) , \end{aligned}$$

where, $E_{S_P}$ is the set of edges that are covered by the pattern *P*, including observed ($weight>0$) and not observed ($weight=0$) interactions.

The measure can be explained through Fig. 1b. Let us consider the subgroup covered by the pattern *Gender=F* $\rightarrow$ *Gender=M* and the interactions between the 5 nodes shown in Fig. 1a. Figure 1cshows the edges considered (a total of 6 edges) for the computation of $q_{\textit{DS}}$: all possible edges, according to nodes’ attributes, including edges between nodes that are not part of a subgroup.

### Attributed balanced multidigraph

If we aim to consider multiple edges between nodes (actors) we can provide a straight-forward extension of the one for the simple digraph. This quality measure is the multidigraph version of $q_{\textit{BD}}$, in Equation 2, making use of the multi-edge information. The quality measure $q_{\textit{BM}}$ can be formalized as follows:

3 $$\begin{aligned} q_{\textit{BM}}(P) = Z\left(\frac{1}{|E_{Mp}|} \cdot \sum _{e \in E_{Mp}} w\left( e\right) \right) \end{aligned}$$

where $E_{Mp}$ is a set of both observed (multi-)edges with $weight>0$ (between every two nodes that interacted, once or more) and not observed edges with $weight=0$ for all two nodes without observed interactions covered by pattern *P*.

### Signed graphs

Some relationships between people can be seen as positive or negative, also in the context of social interactions. Specifically, in some contexts, the positive interactions can be seen as “healthy” interactions and the negative ones as “unhealthy”, e. g., such as bullying in the context of the interactions of children at a playground. If such information about positive and/or negative interaction is available, we can integrate that into a signed graph, essentially by labeling the graph and modifying edges according to the positive and/or negative sign. We can then directly utilize our proposed subgroup discovery approach in order to identify the characteristics of positive or negative interactions.

We start by building a signed graph, $G_{sign}$ based on some attribute *sign*. We may not have information about all edges (e. g., only having information about some positive relationships) and not being positive does not necessarily mean the relationship is negative. Therefore, we only consider either the positive or the negative information. If the edge between two subjects has weight 1 the relationship is positive or negative, respectively, between the two subjects. If the edge has a weight of 0, then we do not assume anything. Then, after computing the interaction graph, *G*, we add the information about the positivity/negativity of the relationship, constructing $G_{sign}$. To do so, we only consider the weights of the edges of the interaction graph for which the corresponding edge in the signed graph is weighted. As such, we can adapt the quality measures above for the case of signed graphs simply by modifying the weight function of the edges. Thus, in any of the quality measures mentioned above, we replace *w*(*e*) by $w_{G}(e)\cdot w_{G_{sign}}(e)\,,$ which can also be precomputed for the signed graph. Finally, we can run the subgroup discovery algorithm with any of the quality measures presented in this section with this simple adaptation.

## Datasets

This section describes the real-world datasets used in our experiments. We distinguish two *types* of social interaction networks: Networks of face-to-face interactions which can be directly formalized as (undirected) social interactions, and spatio-temporal networks from which (directed) social interactions can be derived: (1) two social interaction datasets captured at scientific conferences, i. e., at the LWA 2010 conference in Kassel, Germany (cf.  (Atzmueller et al., 2012)), and the Hypertext (HT) 2011 conference in Eindhoven, The Netherlands (cf.  (Macek et al., 2012)); (2) we utilized datasets capturing interactions (of children) at a playground (Heravi et al., 2018; Messinger et al., 2019), collected with the use of location sensors during the school breaks: *playgroundA* and *playgroundB*. Analyzing social interactions in the playground can be of utmost importance. Social group structure and dynamics are believed to be strongly related to the child well-being and yet has been poorly understood and studied (Heravi et al., 2018). Our software package^2^ which we provide for replication, also contains this as a benchmark dataset.

### Face-to-face interactions: LWA 2010 & HT 2011 datasets

For collecting data at scientific conferences, the Conferator system (Macek et al., 2012) was applied. Conference participants^3^ were invited to wear active RFID proximity tags. 4 When the tags are worn on the chest, tag-to-tag proximity is a proxy for a (close-range) face-to-face (F2F) contact, since the range of the signals is approximately 1.5 m if not blocked by the human body, cf.  (Barrat et al., 2010) for details. This results in time-resolved networks of F2F contacts. Table 1 provides summary statistics of the collected dataset; see (Kibanov et al., 2015) for a detailed description.

In addition to the F2F contacts of the participants, further (socio-demographic) information was available from the Conferator online profile (for LWA 2010), i. e., on the participants’ (1) *gender*, (2) (university) *affiliation*, (3) academic status – *position* – i. e., professor, postdoc, PhD, student, (4) and their main conference *track* of interest. For the HT 2011 dataset, a similar experiment took place. Detailed information about this dataset can also be found in Table 1. This dataset, however, includes information about *country* instead of (university) *affiliation*.

**Table 1:** Statistics/properties of the real-world datasets: number of participants |*V*|, unique contacts |*U*|, total contacts |*C*| average degree, diameter *d*, density, count of F2F contacts (*C*), cf.  (Kibanov et al., 2015) for details

Network	$|V|$	$|U|$	$|C|$	$\varnothing$Degree	*d*	Density
LWA 2010	77	1004	5154	26.08	3	0.34
HT 2011	69	550	1902	15.94	4	0.23

**Table 2:** Statistics/properties of the real-world spatio-temporal datasets: number of social features (#Social), Duration of the data collection, *Duration*, Number of participants, |*V*|, and number of spatial features (#Spatial)

Name	#Social	Duration	|*V*|	#Spatial
PlaygroundA	5	45	18	9
PlaygroundB	2	60	14	2

### Spatio-temporal datasets: playground interactions

For playground interactions, we utilized two different datasets (cf.  Table 2), i. e., the *playgroundA* (Heravi et al., 2018; Gibson et al., 2018), and *playgroundB* (Messinger et al., 2019) datasets, which we describe below in more detail.

The dataset *playgroundA* contains the geographic position of 18 7-8 year-old children (9 girls) over time, during approximately 45min.^5^ It also includes personal attributes (gender, age, social-emotional adjustment etc.). The children were playing outdoors, without toys or playground equipment, during a normal day of Primary School. They had a head-mounted sensor with IMU (Inertial Measurement Unit) and GNSS (Global Navigation Satellite System) for precise positioning a shoe-mounted IMU sensor for activity monitoring. A behaviour rating scale, the Strengths and Difficulties Questionnaire (SDQ,  (Goodman, 2001)), was completed by each child’s class teacher. The questionnaire contains the following sub-scales:

- Social Skills: the higher the score the greater the child’s social skills.
- Conduct Difficulties (Conduct): the higher the score the greater the child’s behavioral problems.
- Emotional Difficulties (Emotion): the higher the score the greater the child’s emotional difficulties.
- Peer Problems (Peer): the higher the score the more difficulty the child has with friendships.
- Hyperactivity (Hyper): the higher the score the greater the child’s level of hyperactivity.

The movement data in this dataset is composed by x and y positions, per time step, and the speed directions in the x and y coordinates. No explicit interactions are given. The latter will be obtained from this movement data. In terms of the personal attributes of this dataset, the numeric values were transformed into 3 equal frequency bins.

The dataset, *playgroundB* (Messinger et al., 2019) has the position of 14 children (8 girls) around 5 years-old and socio-demographic attributes, such as gender and age. The movement data was collected During 1 h using a real time location system referred as UWB (Ultra-Wide Band). Since this dataset did not include speed directions (x and y), this was derived from the positional data.

## Results

In this section, we present our experiments on the datasets described in the previous section, and discuss our results and implications in detail.

### Mining exceptional behavior: datasets on academic conferences

For compositional analysis, we applied subgroup discovery on the attributes described in Sect. 5, utilizing the SD-Map algorithm (Atzmueller and Puppe, 2006), where we supplied our novel quality functions for determining the top-20 subgroups in the datasets LWA 2010 and Hypertext (HT) 2010.

For the target concept, we first investigated the *mean length of contacts* – corresponding to the *duration* of a social interaction in the respective subgroup, focusing on the quality functions $q_{S}$ and $q_{M}$. In addition, we analyzed the *balanced* functions specifically towards homophily and also experimented with variations to using the mean, e. g., applying the variance, which provides for extended statistical characterization, in addition to the significance values which we provide for reference.

**Table 3:** Top-10 most exceptional subgroups according to the aggregated duration of face-to-face interactions at LWA 2010 (simple attributed network): The table shows the respective patterns, the covered number of dyads, the mean interaction length (seconds, $\varnothing$L/S, subgroup and $\varnothing$L/P pattern normalized using quality functions $q_{S}$ and $q_{B}$) and significance compared to the null-model (Z/S, Z/B)

Description	Size	$\varnothing$L/S	$\varnothing$L/P	Z/S	Z/B
track=EQ	456	182.05	410.67	19.01	18.91
affiliation=NEQ	959	245.39	249.92	18.91	18.98
position=NEQ	885	227.44	251.98	17.93	17.93
affiliation=NEQ, position=NEQ	868	220.01	988.00	17.36	4.37
affiliation=NEQ, track=EQ	428	158.18	366.89	16.22	16.29
position=NEQ, track=EQ	392	145.7	367.47	15.71	15.70
gender=NEQ	705	182.5	254.76	15.43	15.35
affiliation=NEQ, position=NEQ, track=EQ	381	139.92	357.69	15.2	15.18
gender=NEQ, track=EQ	312	123.84	371.67	14.01	13.95
affiliation=NEQ, gender=NEQ	669	160.01	227.61	13.2	13.22
gender=NEQ, position=NEQ	627	152.02	233.37	12.89	12.98
affiliation=NEQ, gender=NEQ, position=NEQ	614	145	224.25	12.1	12.34
gender=EQ	299	257.69	321.33	11.91	11.86
gender=EQ, track=EQ	144	189.02	506.47	11.75	12.01
affiliation=NEQ, gender=NEQ, track=EQ	289	102.15	315.73	11.35	11.36
affiliation=NEQ, gender=EQ, track=EQ	139	179.23	491.92	11.25	11.29
affiliation=NEQ, gender=EQ, position=NEQ, track=EQ	120	179.59	507.80	11.13	11.06
gender=EQ, position=NEQ, track=EQ	123	180.46	50161	11.06	11.08
affiliation=NEQ, gender=EQ	290	252.35	306.16	11.01	10.92
affiliation=EQ, track=EQ	28	298.74	1960.50	11.00	10.90

Accordingly, in our experimentation, we applied both simple attributed networks, and multigraph representations: For the former, social interactions between respective actors were aggregated, such that the corresponding weight is given by the sum of all interactions between those actors. For the multigraph case, we considered the face-to-face interactions with their respective duration individually. Tables 3, 4,  5, 6 show the results: We indicate the top-20 patterns for the respective conferences: LWA 2020 in Tables 3, 4 HT 2011 in Tables 5, 6. For both, we show first show the top patterns ranked according to the standard quality functions, while we additionally also include the homophilic versions of the quality functions for comparison. In addition, we provide a detailed view on the important homophilic factors for the multigraphs.

Overall, we notice several common patterns in those tables, both for LWA 2010 and HT 2011: We observe the relatively strong influence of homophilic features such as *gender*, *track*, *country*, and *affiliation* in the detected patterns, confirming preliminary work that we presented in Atzmueller and Lemmerich (2018) only analyzing the individual features and their contribution to establishing social interactions. Using compositional subgroup discovery we can analyze those patterns at a more fine-grained level, also taking more complex patterns, i. e., combinations of different features into account. Thus, our results indicate more detailed findings both concerning the individual durations, the influence of repeating interactions, and the impact of complex patterns given by a combination of several features. We can observe this in Table 3, e. g., regarding the patterns ${gender}={EQ}\,\,\text {AND}\,\, {track}={EQ}$ in correspondance to ${gender}={EQ}\,\,\text {AND}\,\, {posi}={EQ}\,\,\text {AND}\,\, {position}={NEQ}$. Here, we can observe some effect modification in the subgroup; the non-homophilic attribute slightly decrease the subgroup quality (and estimated significance), which we also observe in the respective values, for which the effect for the balanced quality function is more pronounced.

**Table 4:** Detail analysis of homophilic factors w.r.t. the non-aggregated duration of face-to-face interactions at LWA 2010 (attributed multigraph): The table shows the respective patterns, the covered number of dyads, the mean interaction length (seconds, $\varnothing$L/S, subgroup and $\varnothing$L/P pattern normalized using quality functions $q_{S}$ and $q_{B}$) and significance compared to the null-model (Z/S, Z/B)

Description	Size	$\varnothing$L/S	$\varnothing$L/P	Z/S	Z/B
affiliation=EQ	484	123.41	168.05	5.85	5.8
affiliation=NEQ	4670	108.18	109.05	20.94	20.75
gender=EQ	1558	116.26	127.67	11.56	11.28
gender=NEQ	3596	91.8	107.08	13.23	1344
position=EQ	821	107.06	136.99	6.97	696
position=NEQ	4333	104.41	109.29	18.76	18.83
track=EQ	3080	91.09	126.27	13.89	13.85
track=NEQ	2074	77.58	98.04	10.07	10.01

For the compositional multigraph analysis, i. e., focusing on dyadic interactions in the multigraph case, we can observe that this analysis focuses on much more specific patterns; here, this is reflected by higher ratings for more specific patterns. For LWA 2010 and HT 2011 we observe clear indications of homophily, e. g., regarding gender, position, track, and affiliation/country, which is also in line with our expectations, e. g.,  cf.  Atzmueller and Lemmerich (2018), see Tables 4 and 6 for details. More specifically, we both observe *assortativity*, e. g., for the attributes *track*, but also *disassortativity*, e. g., for the attributes *gender* and *position*, regarding the individual rankings when comparing the pattern-induced structures to the respective null-models.

**Table 5:** Top-20 most exceptional subgroups according to the aggregated duration of face-to-face interactions at HT 2011 (simple attributed network): The table shows the respective patterns, the covered number of dyads, the mean interaction length (seconds, $\varnothing$L/S, subgroup and $\varnothing$L/P pattern normalized using quality functions $q_{S}$ and $q_{B}$) and significance compared to the null-model (Z/S, Z/B)

Description	Size	$\varnothing$L/S	$\varnothing$L/P	Z/S	Z/B
gender=EQ	357	114.76	149.80	15.76	15.59
gender=EQ, track=EQ	114	83.87	275.95	15.32	15.14
country=EQ, gender=EQ, track=EQ	35	111.75	893.98	14.21	14.26
country=EQ, track=EQ	42	89.74	622.55	13.89	13.80
track=EQ	185	70.4	174.03	13.73	13.99
country=EQ, gender=EQ, position=NEQ, track=EQ	18	140.52	1,225.90	12.98	12.68
country=EQ, gender=EQ	55	70.06	518.57	12.75	12.84
country=NEQ	470	87.76	95.85	12.61	12.66
country=EQ	80	56.51	393.28	12.59	12.69
position=NEQ	365	76.89	107.40	11.87	11.74
gender=EQ, position=EQ, track=EQ	46	68.43	398.31	11.8	11.55
country=EQ, position=NEQ, track=EQ	23	99.62	724.14	11.62	12.38
position=EQ	185	60.15	149.08	11.45	11.58
position=EQ, track=EQ	60	53.32	270.92	11.44	11.29
country=EQ, gender=EQ, position=NEQ	30	82.03	607.24	11.29	11.16
country=NEQ, gender=EQ	302	82.91	114.65	11.19	11.17
gender=EQ, position=EQ	136	61.91	183.51	10.81	10.99
gender=EQ, position=NEQ	221	71.43	131.05	10.52	10.59
gender=EQ, position=NEQ, track=EQ	68	58.42	214.99	10.13	9.88
track=NEQ	365	70.22	95.27	10.03	9.99
country=EQ, position=NEQ	50	45.89	379.98	9.86	9.72

**Table 6:** Detail analysis of homophilic factors w.r.t. the non-aggregated duration of face-to-face interactions at HT 2011 (attributed multigraph): The table shows the respective patterns, the covered number of dyads, the mean interaction length (seconds, $\varnothing$L/S, subgroup and $\varnothing$L/P pattern normalized using quality functions $q_{S}$ and $q_{B}$) and significance compared to the null-model (Z/S, Z/B)

Description	Size	$\varnothing$L/S	$\varnothing$L/P	Z/S	Z/B
country=EQ	529	42.62	120.36	10.09	10.15
country=NEQ	1373	63.37	67.48	19.74	19.83
gender=EQ	1393	73.28	86.14	20.16	20.08
gender=NEQ	509	33.41	58.52	9.71	9.76
position=EQ	697	47.33	89.21	14.68	14.5
position=NEQ	1205	55.72	70.17	16.05	15.59
track=EQ	832	52.46	94.29	15.75	15.97
track=NEQ	1070	52.85	65.89	14.32	14.15

Regarding the quality measure $q_{B}$ which focuses on the subgraph induced by the pattern, we observe even stronger indications for homophily, as expected. Homophilic interactions should be ranked higher since this quality measure estimates relative to only the possible interactions. Here, for LWA 2010, for example, the pattern ${gender}={EQ}\,\,\text {AND}\,\,{track}={EQ}$ or ${affilitation}={EQ}\,\,\text {AND}\,\,{track}={EQ}$ show strong indications for the aggregated Durations in the simple graph. Likewise, this can also be observed in the multi-graph, for the individual factors regarding their individual interactions, both relating to assortativity and disassortativity as discussed above. Similar observations can be made for HT 2011, e. g., for the pattern ${country}={EQ}\,\,\text {AND}\,\,{gender}={EQ}\,\,\text {AND}\,\,{track}={EQ}$ while there are also some refinements which consider a non-equal position. The individual characteristics of the homophilic factors for HT 2011 can be observed in Table 6 for the multi-graph case, which reflects the patterns for the aggregated behavior (Tables 3, 4, 5), however, on the level of individual interactions.

Overall, we observe that the quality measure $q_{B}$ confirms the assessment of the individual quality measures $q_{S}$ and $q_{M}$ which focus on the simple and aggregated graphs – enabling a comparison in terms of the sizes of the subgroup. Quality measure $q_{B}$ focuses more on the specifics of the pattern, taking its semantics for normalizing the mean into account. While the patterns based on the mean quality functions allow a simple assessment, we also experimented with variants taking into account the variance of the respective parameters. Overall, there were no considerable differences in the obtained top subgroup patterns, stressing the importance of the homophilic factors shown in our results tables presented above. Therefore, both measures provide complementing perspectives; regarding mean/variance, the mean is often more interpretable but the variance can supply additional statistical insights into the analysis, while the statistical significance is transparently estimated by our evaluation and assessment approach.

### Mining exceptional behavior on socio-spatial data: PlaygroundA

In the following sections, we analyze the *playgroundA* data regarding exceptional behavior on the socio-spatial characteristics. First, we consider the simple digraph and multidigraph cases using the quality functions normalizing the behavior w.r.t. the nodes of the individual induced subgraphs (i. e., applying quality measures $q_{\textit{DS}}$ and $q_{\textit{DM}}$). Here, we also consider variance-based variants, in addition to the standard mean-based quality functions. After that, we consider the homophilic features in more detail, considering only the possible interactions for our null-model according to the pattern semantics on homophily (i. e., using quality measures $q_{\textit{BD}}$ and $q_{\textit{BM}}$), before we consider the analysis of the signed graphs.

#### Baseline analysis: exceptional behavior – quality measures $q_{\textit{DS}}$ and $q_{\textit{DM}}$

For reference to the discussion in the following section, Table 7 shows the respective top-3 ranked subgroups found in the dataset *playgroundA*, with quality measure $q_{SM}$ in three different versions: a comparison version, a to-node and from-node version with attributed multidigraph version (comp, to and from in the column V, in the Table 7, respectively), building on our presentation in Centeio Jorge et al. (2019, 2021). Basically, these results apply the standard quality functions for compositional subgroup analysis, which serve as a baseline for our directed networks, comparing these to the homophilic quality functions for which we discuss their results below.

Essentially, for each subgroup, we show its pattern, number of nodes (children) belonging to the subgroup, *N*, number of edges (interactions), *E*, the mean time of an interactions between children in the subgroup, |*C*|, and the Z-score based on the comparison between the total duration of the interactions in the subgroup and the null model, *Z*.

**Table 7:** Reference ranking of subgroups (comp, to-node and from-node attributed multidigraph): total duration of interactions between every two children in dataset *playgroundA* with quality measure $q_{\textit{DM}}$, cf.  (Centeio Jorge et al., 2019, 2021)

Rank	V	Pattern	*N*	*E*	|*C*|	*Z*
1	comp	Gender=M $\rightarrow$ Gender=M $\wedge$ Age=same $\rightarrow$ Age=same $\wedge$ Social_Skills=same $\rightarrow$ Social_Skills=same	5	176	3.1	195.3
2	comp	Gender=M $\rightarrow$ Gender=M $\wedge$ Emotion=same $\rightarrow$ Emotion=same $\wedge$ Social_Skills=same $\rightarrow$ Social_Skills=same	5	162	2.9	179.1
3	comp	Age=same $\rightarrow$ Age=same$\wedge$ Emotion=same $\rightarrow$ Emotion=same $\wedge$ Social_Skills=same	6	110	2.1	153.7
1	to	Age=medium $\wedge$ Gender=F $\wedge$ Conduct=high $\wedge$ Social_Skills=high $\wedge$ Emotion=high $\wedge$ Peer=medium	12	156	0.5	21.4
2	to	Conduct=high $\wedge$ Gender=F $\wedge$ Emotion=high $\wedge$ Hyper=low $\wedge$ Peer=medium	12	156	0.5	21.4
3	to	Conduct=high $\wedge$ Social_Skills=high $\wedge$ Emotion=high $\wedge$ Peer=medium	12	156	0.5	21.4
1	from	Conduct=high $\wedge$ Social_Skills=high	12	174	0.4	14.5
2	from	Conduct=high $\wedge$ Gender=F $\wedge$ Emotion=high $\wedge$ Hyper=low $\wedge$ Peer=medium	12	174	0.4	13.7
3	from	Age=medium $\wedge$ Gender=F $\wedge$ Conduct=high $\wedge$ Social_Skills=high $\wedge$ Emotion=high $\wedge$ Peer=medium	12	174	0.4	13.7

As we can observe, the top ranked subgroups (Table 7) obtained with the comparison version include social attributes as *same*, such as $Gender=M \rightarrow Gender=M$, $Gender=F \rightarrow Gender=F$, $Age=same \rightarrow Age=same$ and $Social\_Skills=same \rightarrow Social\_Skills=same$. Here, children with the same social attributes’ values interact and follow each much more than what would be expected (Centeio Jorge et al., 2021). This is also in line with the observations already discussed in Messinger et al. (2019). Overall, these observations seem to confirm the homophily hypothesis with respect to the set of attributes as mentioned above, in particular taking into account the gender, meaning that children interact more with children of the same gender.

**Table 8:** Reference ranking of subgroups (comp, to-node and from-node attributed digraph): total duration of interactions between every two children in the dataset *playgroundA* with quality measure $q_{\textit{DS}}$, cf.  (Centeio Jorge et al., 2019, 2021)

Rank	V	Pattern	*N*	*E*	|*C*|	*Z*
1	comp	Gender=F $\rightarrow$ Gender=F $\wedge$ Emotion=same $\rightarrow$ Emotion=same $\wedge$ Hyper=same $\rightarrow$ Hyper=same	4	12	64.1	42.8
2	comp	Gender=M $\rightarrow$ Gender=M	8	39	36.3	33.9
3	comp	Gender=F $\rightarrow$ Gender=F	8	54	35.6	32
1	to	Peer=low	16	122	18	5.2
2	to	Gender=M $\wedge$ Peer=low	16	67	11.6	5.6
3	to	Gender=M $\wedge$ Social_Skills=medium	13	21	7.5	5.1
1	from	Peer=low	16	123	18	6.2
2	from	Gender=M $\wedge$ Peer=low	16	70	11.4	5.1
3	from	Conduct=low $\wedge$ Social_Skills=medium $\wedge$ Peer=low	13	21	7.2	4.9

Likewise, we also show the top-3 comparison, to-node and from-node versions of the quality measure for attributed directed graphs, $q_{SD}$ in Table 8, as our baseline result. Unlike $q_{SM}$, $q_{SD}$ does not take into account the frequency of interactions, focusing on the duration of interactions. In contrast, we only consider the total duration of a (directed) interaction between pairs of children. As we can observe, the results of the *comp* version are similar to the multidigraph version. The best ranked subgroups also show homophily, especially regarding gender, while other attributes that seem to be significant when shared between children are *Emotion*, *Hyper* and *Peer* (Centeio Jorge et al., 2021). So, overall, we also observe homophily, especially regarding gender.

Furthermore, for all versions of the applied quality functions, we computed the quality measures using variance instead of mean duration of interactions of each subgroup. The results were very similar. The Z-score, however, appears to be show more inter-space in the variance versions.

#### Exceptional homophilic analysis – quality measures $q_{\textit{BD}}$ and $q_{\textit{BM}}$

**Fig. 2:**
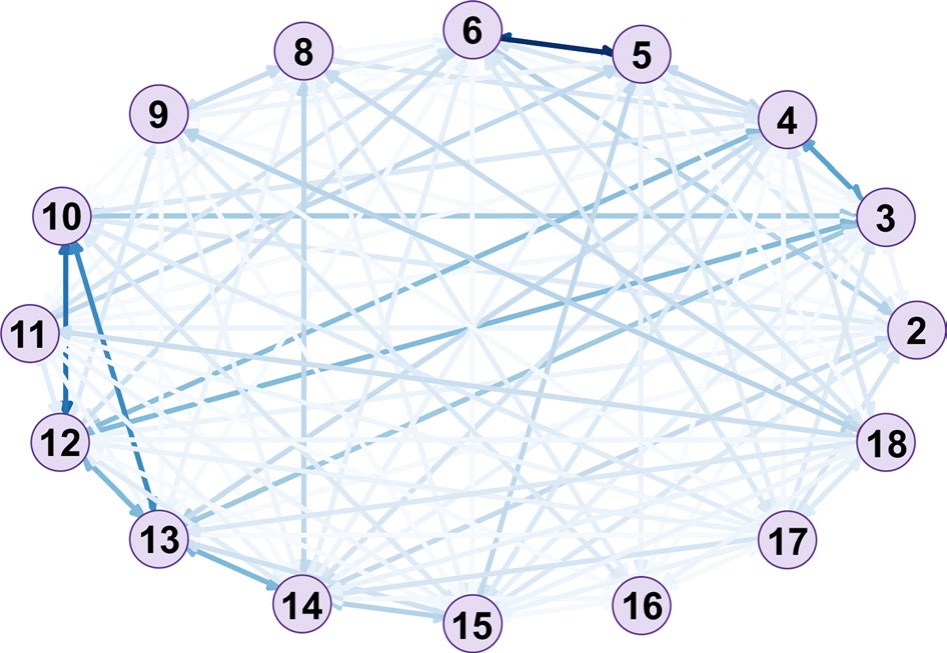
Complete graph of interactions in *playgroundA*

For a more complete and better analysis of the results presented in the previous section, we present results of some experiments with the quality measures $q_{\textit{BD}}$ and $q_{\textit{BM}}$. For these measure, we consider both digraph and multidigraph structures, *comp*, *to-node*, and *from-node* versions and mean and variance as metrics to consider for the computation of the quality of the subgroups. Furthermore, since these quality measures take into account all edges between all nodes, we can also analyse the lowest values of the Z-score. This gives us insights regarding the subgroups that show unexpectedly *short* duration of interaction.

**Fig. 3:**
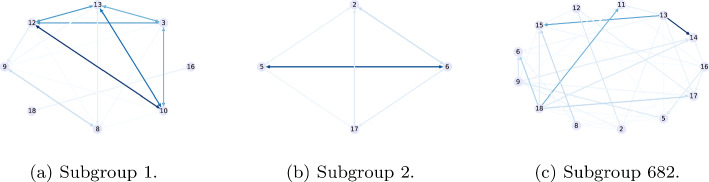
Subgraphs representing subgroups (**a**) 1 (**b**) 2 and (**c**) 682 from Table 9

We start by presenting the results of $q_{\textit{BD}}$, for *comparison version*, using *mean* as metric in Table 9. This table presents both the subgroups with highest and lowest Z-scores. The subgraphs correspondent to the subgroups with highest score, *Gender=M* $\rightarrow$ *Gender=M* $\wedge$ *Peer=same* $\rightarrow$ *Peer=same* and *Gender=F* $\rightarrow$ *Gender=F* $\wedge$ *Emotion=same* $\rightarrow$ *Emotion=same* $\wedge$ *Hyper=same* $\rightarrow$ *Hyper=same*, can be seen in Fig. 3a and b, respectively. On the other hand, Fig. 2 shows the complete graph of interactions between all children in *playgroundA*. The darker the color of the edge, the higher the weight it presents. We can see some of the darker edges show the interactions between the nodes 6 and 5, a triangle among nodes 10, 12 and 13. In the figures showing the subgroups, we can see the first subgroup includes the characteristics that the nodes 10, 12 and 13 have in common. Additionally, it shows us that the node 3 has also strong interactions with these nodes and shares the characteristics. Moreover, subgroup 2 succeeds in capturing the characteristics of the edge between nodes 6 and 5, the darker in the graph of interactions. Finally, the subgroup with the lowest Z-score is shown in Fig. 3c. We can see that its heaviest edge (from node 13 to node 14), is very light in the complete interactions graph. It is, indeed, unexpected to see these weights in a subgroup, in the sense that they are all very low.

**Table 9:** Ranking of subgroups (*comparison* attributed digraph version) according to the *mean* of the total duration of interactions between every two children in the dataset *playgroundA* for quality measure $q_{\textit{BD}}$

Rank	Pattern	*N*	*E*	|*C*|	*Z*
1	Gender=M $\rightarrow$ Gender=M $\wedge$ Peer=same $\rightarrow$ Peer=same	8	32	50.1	4
2	Gender=F $\rightarrow$ Gender=F $\wedge$ Emotion=same $\rightarrow$ Emotion=same $\wedge$ Hyper=same $\rightarrow$ Hyper=same	4	12	64.1	3.6
3	Gender=F $\rightarrow$ Gender=F $\wedge$ Emotion=same $\rightarrow$ Emotion=same	8	16	55.9	3.5
4	Gender=M $\rightarrow$ Gender=M $\wedge$ Social=same $\rightarrow$ Social=same $\wedge$ Conduct=same $\rightarrow$ Conduct=same	7	10	64.3	3.4
5	Gender=F $\rightarrow$ Gender=F $\wedge$ Conduct=same $\rightarrow$ Conduct=same	8	14	57.4	3.3
.	.	.	.	.	.
678	Gender=M $\rightarrow$ Gender=F $\wedge$ Hyper=lower $\rightarrow$ Hyper=higher $\wedge$ Social=higher $\rightarrow$ Social=lower $\wedge$ Emotion=higher $\rightarrow$ Emotion=lower	11	27	11.1	$-$2.4
679	Gender=M $\rightarrow$ Gender=F $\wedge$ Hyper=lower $\rightarrow$ Hyper=higher	13	38	13.6	$-$2.5
680	Gender=F $\rightarrow$ Gender=M $\wedge$ Hyper=higher $\rightarrow$ Hyper=lower	13	38	13	$-$2.5
681	Gender=F $\rightarrow$ Gender=M $\wedge$ Hyper=higher $\rightarrow$ Hyper=lower $\wedge$ Social=lower $\rightarrow$ Social=higher	13	37	12.5	$-$2.6
682	Gender=M $\rightarrow$ Gender=F $\wedge$ Hyper=lower $\rightarrow$ Hyper=higher $\wedge$ Social=higher $\rightarrow$ Social=lower	13	37	12.6	$-$2.7

**Fig. 4:**
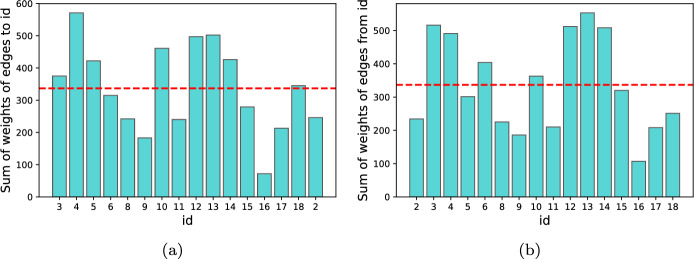
Sum of edge weights: (**a**) leading to nodes by id; (**b**) starting at node by id

In Table 10 we show results for $q_{\textit{BD}}$, using variance as metric in a *from-node* version. Using variance as metric in this quality measure can also achieve very good results. For example, the first subgroup ranked in the list includes two “out-nodes", meaning that the edges are directed to two nodes. In this case, these nodes have ids 6 and 10. Figure 4b shows, in a bar plot, the sum of the weights of edges directed to each id. In fact, both nodes 6 and 10 are above average. The second subgroup presents less detailed characteristics and includes, besides nodes 6 and 10, nodes 12 and 14, that also present very high sum of weights of the edges directed to them. On the other hand, the subgroup lowest in this rank, *Gender=F *$\wedge$ *Social=high *$\wedge$ *Conduct=low *$\wedge$ *Hyper=low*, is composed of the out-nodes 2, 15 and 17, all below average in the bar plot presented in Fig. 4b.

**Table 10:** Ranking of subgroups (from-node attributed digraph version) according to the variance of the total duration of interactions between every two children in the dataset *playgroundA* for quality measure $q_{\textit{BD}}$

Rank	Pattern	*N*	in	out	*E*	|*C*|	*Z*
1	Social=medium $\wedge$ Peer=low $\wedge$ Hyper=low	16	16	2	30	2890.3	2.5
2	Social=medium $\wedge$ Peer=low	16	16	4	60	2234.7	2.2
3	Social=medium $\wedge$ Gender=M $\wedge$ Conduct=low	16	16	2	30	2619.7	2.2
.	.	.	.	.	.	.	.
1443	Gender=F $\wedge$ Social=high $\wedge$ Conduct=low $\wedge$ Emotion=high $\wedge$ Hyper=low	16	16	2	30	246.5	$-$1.6
1444	Gender=F $\wedge$ Social=high $\wedge$ Conduct=low $\wedge$ Hyper=low	16	16	3	45	308.4	$-$1.8

Similarly, Table 11, shows the *to-node* version of $q_{\textit{BD}}$ and Fig. 4a shows, in a bar plot, the sum of the weights of the edges starting at each node by id. The first ranked subgroup shows the characteristics of two *to-nodes*, with ids 10 and 12, that also show a very high value in the bar plot of Fig. 4a. Alternatively, the subgroup with the lowest Z-score is composed of nodes 2, 15 and 17, all showing values lower than average in the bar plot. It is very interesting to see that the *comparison*, *from-node* and *to-node* are very consistent and aligned in this dataset and quality measure. We can see that some nodes are constantly in high ranked subgroups, such as node 10 and 12, whereas some show unexpected short period of interactions both in *to-node* and *from-node* versions, such as 2, 15 and 17.

**Table 11:** Ranking of subgroups (to-node attributed digraph version) according to the *variance* of the total duration of interactions between every two children in the dataset *playgroundA* for quality measure $q_{\textit{BD}}$

Rank	Pattern	*N*	in	out	*E*	|*C*|	*Z*
1	Social=medium $\wedge$ Gender=M $\wedge$ Conduct=low $\wedge$ Peer=low	16	2	16	30	2733.8	2.2
2	Gender=F $\wedge$ Hyper=low $\wedge$ Conduct=high	16	2	16	30	2657.7	2.1
3	Social=medium $\wedge$ Peer=low	16	4	16	60	2144.9	2
.	.	.	.	.	.	.	.
1442	Emotion=high $\wedge$ Conduct=low $\wedge$ Hyper=low $\wedge$ Gender=F $\wedge$ Social=high	16	2	16	30	303.1	$-$1.5
1443	Age=low	16	7	16	105	855.1	$-$1.6
1444	Gender=F $\wedge$ Social=high $\wedge$ Conduct=low $\wedge$ Hyper=low	16	3	16	45	326.8	$-$1.8

**Fig. 5:**
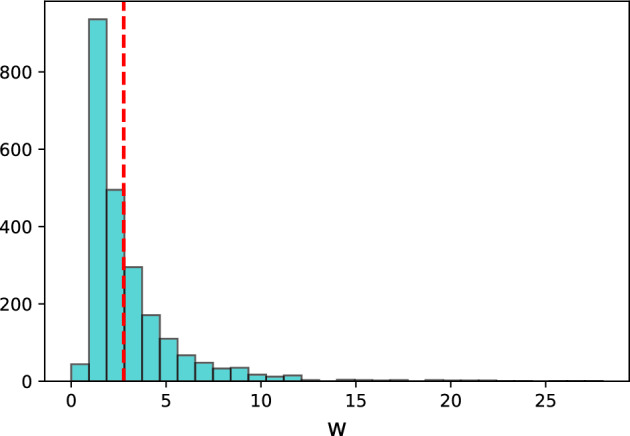
Histogram showing the distribution of the edges’ weights in a complete multidigraph of interactions of *playgroundA*

In Table 12, we present the results of the *comparison* version, with mean being used as metric for the multidigraph quality measure $q_{\textit{BM}}$. Since it is quite hard to represent the weights of multidiedges in a plot, we will use the histogram of edges’ weights in the complete multidigraph, shown in Fig. 5. The average weight of an edge in this complete multidigraph of interactions is 2.76 and the most incident bin of values being between 1 and 2. Furthermore, we can observe that the subgroup with highest subgroup shows a mean value of 3.3 with 130 edges overall. More specifically, this subgroup, *Social=lower* $\rightarrow$ *Social=higher* $\wedge$ *Age=lower* $\rightarrow$ *Age=higher* $\wedge$ *Hyper=higher* $\rightarrow$ *Hyper=lower* $\wedge$ *Gender=M* $\rightarrow$ *Gender=F*, represents interactions from younger boys with lower social skills and more problems with hyperactivity towards older girls with higher social skills and less problems with hyperactivity.

It is very interesting to see that multidigraph quality measure $q_{\textit{BM}}$, which considers not only the weights of the edges, but also their multiplicity (interpreted as frequency of interaction) does not classify homophily as interesting as other measures. In fact, the subgroup covered by the pattern *Gender=F* $\rightarrow$ *Gender=M* is still very high on the rank and the subgroup covered by the pattern *Gender=M* $\rightarrow$ *Gender=M* is the lowest in the rank. This is due to high frequency of interactions – and, consequently, this is accompanied by both a low mean and and a low Z-score. Also, this finding was the same for the version where we used variance as metric in the respective quality measure.

We can conclude that all quality measures find interesting subgroups but treat homophily differently. The quality measure $q_{\textit{BM}}$ shows little homophily, including Gender homophily, in the high-scored subgroups. In both $q_{\textit{BD}}$ and $q_{\textit{BM}}$, the findings when using *variance* and *mean*, as metric for the computation of quality measure, were rather similar and nicely complemented each other. The use of the *mean* as metric, however, facilitates the interpretation of the values.

**Table 12:** Ranking of subgroups (comparison attributed multidigraph version) according to the *mean* of the total duration of interactions between every two children in the dataset *playgroundA* for quality measure $q_{\textit{BM}}$

Rank	Pattern	*N*	in	out	*E*	|*C*|	*Z*
1	Social=lower $\rightarrow$ Social=higher $\wedge$ Age=lower $\rightarrow$ Age=higher $\wedge$ Hyper=higher $\rightarrow$ Hyper=lower $\wedge$ Gender=M $\rightarrow$ Gender=F	11	6	5	130	3.3	2.7
2	Peer=same$\rightarrow$Peer=same $\wedge$ Hyper=higher $\rightarrow$ Hyper=lower $\wedge$ Gender=M $\rightarrow$ Gender=F	12	6	6	101	3.5	2.6
3	Age=lower $\rightarrow$Age=higher $\wedge$ Hyper=higher $\rightarrow$ Hyper=lower $\wedge$ Gender=M $\rightarrow$ Gender=F	11	6	5	130	3.3	2.6
.	.	.	.	.	.	.	.
9	Gender=F $\rightarrow$ Gender=M	16	8	8	403	3	2.1
.	.	.	.	.	.	.	.
713	Emotion=lower $\rightarrow$ Emotion=higher $\wedge$ Peer=lower $\rightarrow$ Peer=higher $\wedge$ Gender=M $\rightarrow$ Gender=F	9	3	6	127	2.1	−3
714	Social=lower $\rightarrow$ Social=higher $\wedge$ Emotion=lower $\rightarrow$ Emotion=higher $\wedge$ Peer=lower $\rightarrow$ Peer=higher $\wedge$ Gender=M $\rightarrow$ Gender=F	9	3	6	127	2.1	−3
715	Gender=M $\rightarrow$ Gender=M	8	8	8	790	2.6	−3

#### Signed graphs and quality measures $q_{\textit{BD}}$ and $q_{\textit{BM}}$

**Fig. 6:**
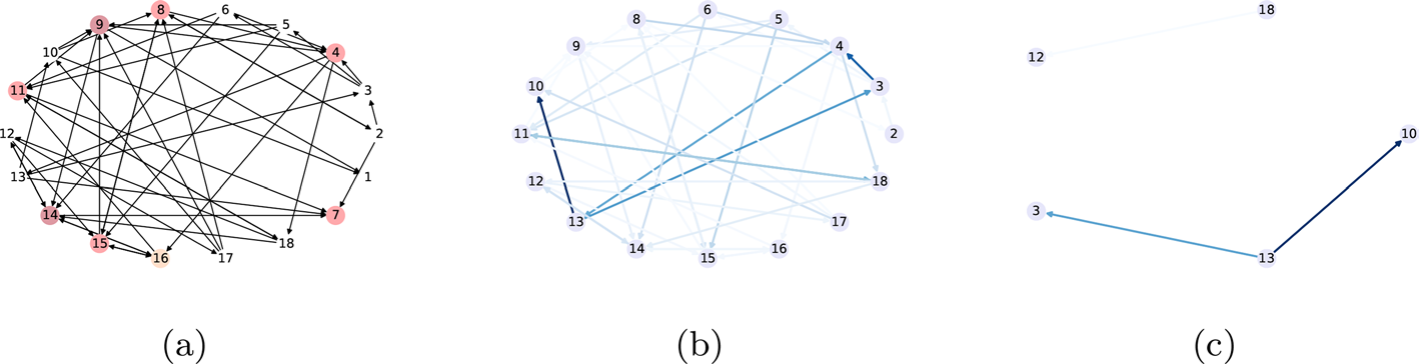
**a** Directed graph showing who dislikes who. **b** Complete negative interactions. **c** Subgroup 1 in $q_{\textit{BD}}$ for negative interactions

The *playgroundA* dataset has the information, for each child, of three peers they nominated as “less liked"by them. As such, we represent this information as a graph in Fig. 6a. We assume that if a child says they do not like another child and still approach them, this is a negative interaction. This should be taken into account and it can mean some bullying or violence of some kind. Figure 6b shows the negative interactions.

The comparison version with mean as metric for quality measure $q_{\textit{BD}}$ ranks the subgroup presented in Fig. 6c as the one with highest quality. This subgroup is covered by the pattern *Age=lower* $\rightarrow$ *Age=higher* $\wedge$ *Gender=M* $\rightarrow$ *Gender=M* $\wedge$ *Hyper=higher* $\rightarrow$ *Hyper=lower* and includes edges from 13 to the nodes 10 and 3, and one edge from the node 18 to the node 12. By analysing Fig. 6c, we see that the edges from node 13 to nodes 10 and 3 and from node 18 to node 12 and very dark, meaning their weights are high. We can conclude that this quality measure successfully finds patterns that cover these exceptional interactions. Furthermore, the versions *from-node* ranked as highest quality subgroups with patterns that cover the characteristics of node 13. The *to-node* version shows that nodes 4 and 10 share several characteristics and that they receive unexpected negative interactions. In this case, both digraph and multidigraph presented similar results, as well as using *mean* or *variance* as metrics in the computation of the quality measures.

### Mining exceptional behavior on socio-spatial sata: PlaygroundB

We also used quality measures $q_{\textit{BD}}$ and $q_{\textit{BM}}$ to analyze the dataset *playgroundB*. In table 13, we show the results of $q_{\textit{BD}}$ using *mean* as metric for comparison version on the digraph of the interactions in the playground. It is very interesting to see that all top-scored subgroups are covered by patterns that show homophily regarding gender, including *Gender=M* $\rightarrow$ *Gender=M* and *Gender=F* $\rightarrow$ *Gender=F*. On the other hand, the two lowest ranked subgroups show exactly the opposite, *Gender=M* $\rightarrow$ *Gender=F* and *Gender=F* $\rightarrow$ *Gender=M*.

**Fig. 7:**
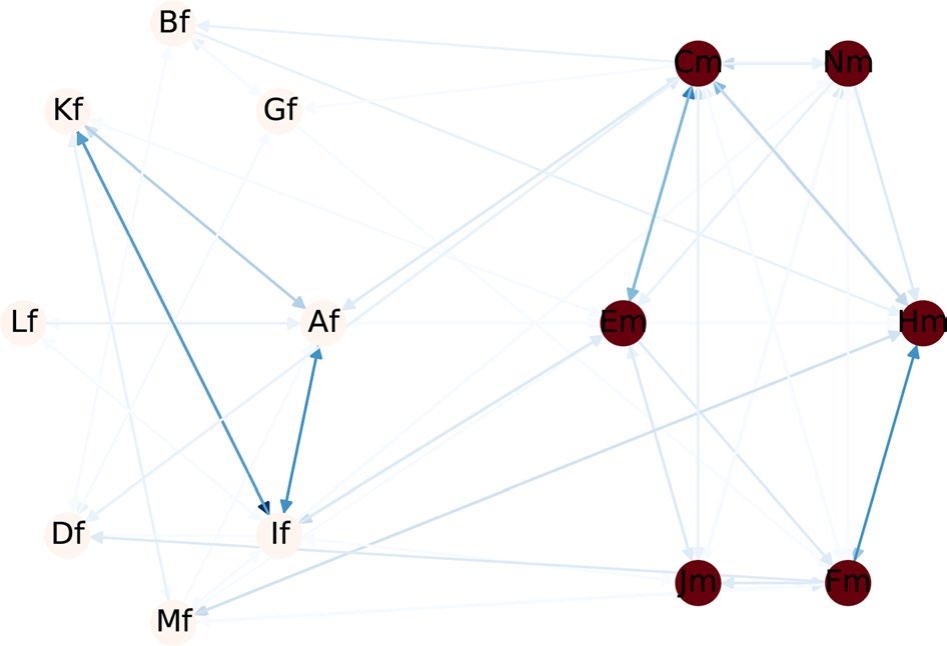
Interactions in *playgroundB* dataset. The nodes (indicating children) are represented in two positional and differently colored clusters: the lighter nodes represent girls and darker nodes represent boys. The darker the color of the edge, the heavier (higher) the weight

These results go according to the main findings of the paper that presented this dataset (Messinger et al., 2019). The authors studied how kindergarten children tend to form gender-segregated, transitive social ties. For example, they mention how by 4-5 years of age, both boys and girls interact with same-sex peers three to four times more often than with other-sex peers (Martin et al., 2013). Furthermore, the interactions digraph in Fig. 7 shows the directed graph of interactions between all children present in the dataset. For the sake of presentation, we show two clusters, a lighter colored one (on the left) whose nodes represent girls, and a darker colored one (on the right) whose nodes represent boys. We can see that, in fact, there are longer interactions (darker edges) inside the clusters.

**Table 13:** Ranking of subgroups (comparison attributed digraph version) according to the *mean* of the total duration of interactions between every two children in the dataset *playgroundB* for quality measure $q_{\textit{BD}}$

Rank	Pattern	*N*	in	out	*E*	|*C*|	*Z*
1	Gender=M $\rightarrow$ Gender=M	6	6	6	30	3.2	4.4
2	Gender=M $\rightarrow$ Gender=M $\wedge$ Age=lower $\rightarrow$ Age=higher	6	4	4	12	4	3.4
3	Gender=F $\rightarrow$ Gender=F $\wedge$ Age=same $\rightarrow$ Age=same	8	8	8	14	2.7	2.1
4	Gender=M $\rightarrow$ Gender=M $\wedge$ Age=higher $\rightarrow$ Age=lower	6	4	4	12	2.9	2.1
5	Gender=M $\rightarrow$ Gender=M $\wedge$ Age=same $\rightarrow$ Age=same	6	6	6	6	2	1
.	.	.	.	.	.	.	.
18	Gender=M $\rightarrow$ Gender=F	14	8	6	48	0.6	−2.1
19	Gender=F $\rightarrow$ Gender=M	14	6	8	48	0.5	−2.5

## Conclusions

In this paper, we presented a method for compositional subgroup discovery, i. e., for detecting interesting compositional patterns in attributed networks capturing dyadic relations. Their interestingness is formalized via specific quality measures, for which we presented seven novel measures in the scope of the dyadic setting. The adapted approach, based on subgroup discovery and exceptional model mining methods (Atzmueller, 2015; Duivesteijn et al., 2016; Atzmueller et al., 2016) enables the identification of exceptional social behavior on attributed interaction networks.

For our presented approach, we adapted principles of subgroup discovery, as a general data analysis technique for exploratory data mining – to the dyadic network setting enabling compositional subgroup discovery. This enables analysis both for social interaction networks as well as socio-spatial interactions including directed and signed networks. Given network structure as well as attributes – which can also be enriched using network science metrics – this can be formalized as an attributed interaction network.

Our results indicate interesting findings w.r.t. common principles observed on social interactions. The influence of homophily and homophilic features on the interactions, e. g., regarding face-to-face interactions at academic conferences can be observed. Furthermore, this is also apparent regarding the PlayGroundA and PlayGroundB datasets, concerning children playing in school playgrounds. The patterns were also rather explainable and interpretable for a domain specialist. Here, results for the signed graphs demonstrated interesting interpretable insights. Specifically, for the playgroundb dataset, this proved very interesting since the paper in which this dataset is explored originally, (Messinger et al., 2019) states the existence of this phenomenon of homophily. Enriching the dataset with network science metrics also showed to give extra insights of the scenario.

The proposed quality functions enable a focused analysis on specific properties of interest according to the applied modeling method. This concerns, in particular, whether a simple attributed network or a multigraph representation is applied. In addition, the proposed quality functions are statistically well-founded, and provide a statistical significance value directly, also easing their interpretation.

For future work, we aim to extend the concepts developed in this work towards feature-rich complex network approaches, e. g.,  (Scholz et al., 2013; Kanawati, 2015; Interdonato et al., 2019), also considering further (large-scale) datasets.^6^

## Data Availability

Our released software package also contains benchmark data.

## Footnotes

1 https://pypi.org/project/graph-sd/

2 https://pypi.org/project/graph-sd/

3 Study participants also gave their informed consent for the use of their data (including their profile) in scientific studies.

4 http://www.sociopatterns.org

5 Ethical approval for this study was gained from the institutional ethics committee. As participants were minors, informed consent to participate was received from those with legal caregiving responsibility for each child. Assent to participate was checked with each child before data collection.

6 https://pypi.org/project/graph-sd/.

## Code availability

We release our software package$^{6}$ demonstrating the presented approach.
